# Multi-agent AI toward autonomous design of perovskite solar cells

**DOI:** 10.1016/j.xinn.2026.101596

**Published:** 2026-09-30

**Authors:** Yongchang Liu, Lijie Zheng, Nursultan Mussakhanuly, Zhong Chen, Jessica Yajie Jiang, Jianghui Zheng

**Affiliations:** 1 School of Electronic Science and Engineering, Xiamen University, Xiamen 361005, China; 2 Shenzhen Research Institute of Xiamen University, Shenzhen 518057, China; 3 Institute of New Materials and Energy Technologies, Nazarbayev University, Astana 010000, Kazakhstan; 4 School of Photovoltaic and Renewable Energy Engineering, University of New South Wales, Sydney, NSW 2052, Australia

## Main text

Metal halide perovskite solar cells (PSCs) have attracted considerable attention over the past decade owing to their outstanding optoelectronic properties and compatibility with low-temperature solution processing.^1^ With certified single-junction PSC efficiencies now reaching 28.0%, further progress hinges on improving operational stability rather than pursuing efficiency gains alone. Unlike power conversion efficiency, operational stability is governed by multiple degradation processes that evolve under thermal, electrical, and illumination stresses, which makes overall optimization substantially more challenging. This complexity has stimulated growing interest in the use of artificial intelligence (AI) for materials discovery and photovoltaic engineering. In particular, AI-assisted approaches capable of integrating knowledge across multiple functional layers are emerging as an important direction in next-generation perovskite photovoltaics.

## Limits of isolated optimization

Most AI-assisted studies of PSCs still rely on predictive machine learning (ML) models to optimize individual materials or interfaces. These approaches have substantially accelerated the discovery of promising perovskite compositions, charge-transport materials, and interface modifiers.^2^ However, they generally treat each functional layer as an independent optimization target, overlooking the strong coupling among absorber chemistry, transport layers, buried interfaces, and overall device architecture. As a result, improvements achieved for individual components often fail to translate into optimal device performance when multiple degradation pathways interact.

This limitation becomes particularly evident in the design of PSCs with long-term stability (Figure 1A). A perovskite composition with reduced bulk defects may not be compatible with a specific transport layer, while an interface modification that enhances charge extraction may compromise long-term thermal or ultraviolet stability. Likewise, suppressing one degradation pathway can inadvertently accelerate another by altering interfacial energetics or ion migration. These trade-offs reflect the inherently coupled nature of PSC degradation and highlight the limitations of isolated optimization. Achieving durable PSCs therefore requires coordinated design across multiple functional layers rather than sequential optimization of individual components.

**Figure 1:**
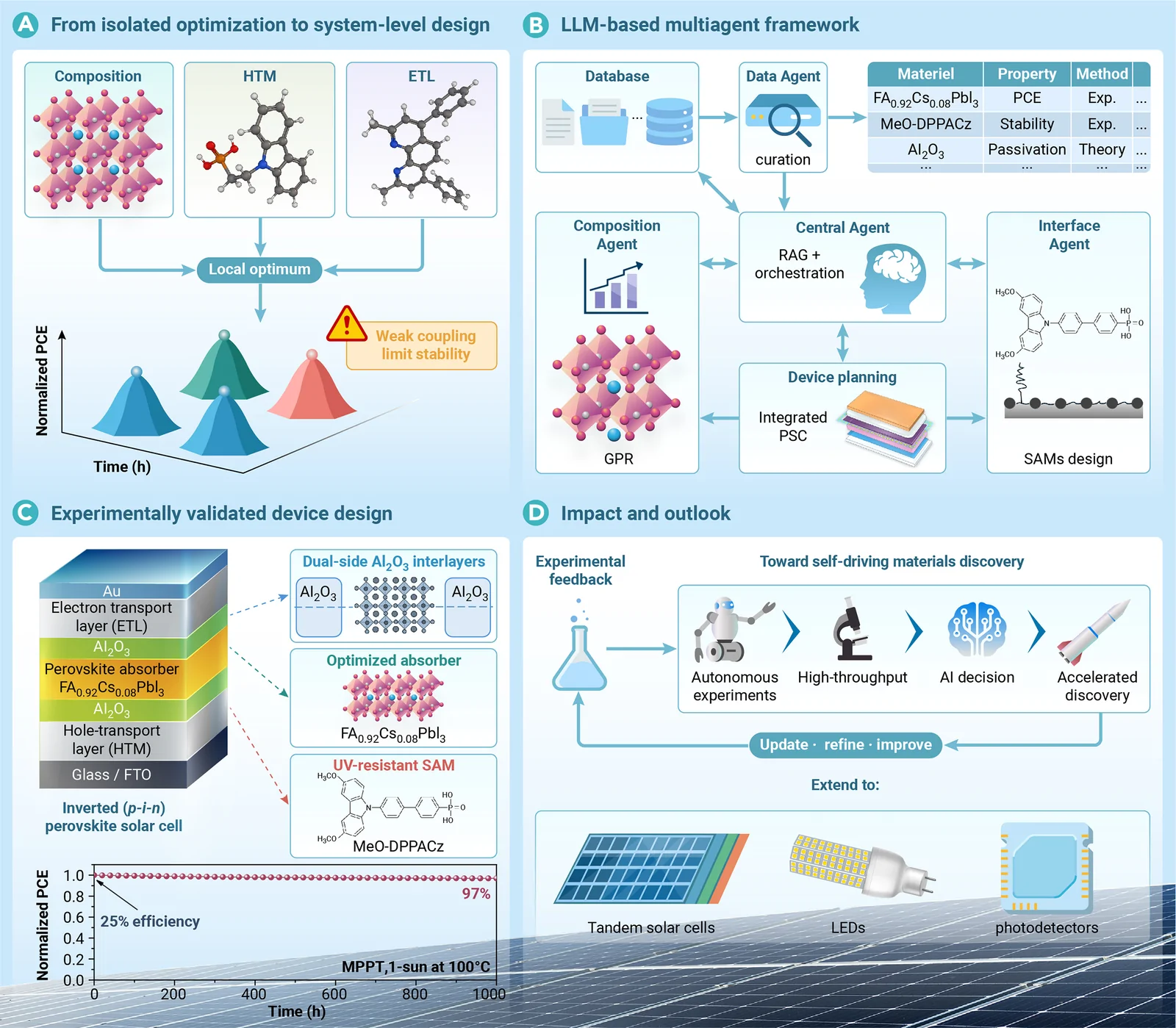
Conceptual transition from isolated materials optimization to LLM-enabled multi-agent co-design of perovskite solar cells (A) Schematic illustration of component-level optimization, in which perovskite compositions, hole-transport materials (HTMs), and electron-transport layers (ETLs) are independently optimized. The individual performance peaks represent local optima, while the conceptual performance–time profiles highlight the potential limitations of weakly coupled optimization for long-term device stability. (B) Architecture of the large-language-model (LLM)-based multi-agent framework, comprising a data curation agent, a central reasoning agent, and specialized agents for composition optimization and interface design. Bidirectional arrows indicate information exchange and task coordination, supported by retrieval-augmented generation (RAG). Gaussian process regression (GPR) is employed for composition optimization, while molecular design informs interface engineering. (C) Experimentally validated inverted (p-i-n) perovskite solar cell integrating an optimized FA_0.92_Cs_0.08_PbI_3_ absorber, dual-sided Al_2_O_3_ interlayers, and a UV-resistant MeO-DPPACz self-assembled monolayer. The corresponding stability profile shows over 97% retention of the initial power conversion efficiency after 1,000 h of maximum-power-point tracking under 1-sun illumination at 100°C, with an initial efficiency of 25.0%. (D) Prospective evolution toward self-driving materials discovery through the integration of autonomous experimentation, high-throughput characterization, AI-guided decision-making, and iterative experimental feedback. The framework illustrates potential extensions to tandem solar cells, LEDs, and photodetectors.

## Collaborative reasoning

A central advance of the work by Guo et al. lies in the introduction of a collaborative reasoning framework for materials discovery.^3^ Rather than relying on a single predictive model, the authors developed a large-language-model (LLM)-based multi-agent system that distributes complementary scientific tasks among specialized agents while coordinating them toward a shared device objective (Figure 1B). Within this architecture, the data agent extracts and structures knowledge from unstructured scientific literature, providing a knowledge base for subsequent reasoning. Building on this knowledge base, the central reasoning agent integrates information from different sources, identifies key stability challenges, and decomposes the complex design problem into specific optimization tasks. These tasks are subsequently assigned to specialized agents, including the composition agent for quantitative optimization of absorber compositions and the interface agent for molecular interface design, enabling coordinated optimization across different aspects of photovoltaic device design.

Despite these conceptual advantages, such information flow also presents potential reliability challenges. LLM-based literature mining by the data agent may introduce hallucinated or inaccurate information, particularly when interpreting experimental conditions or extracting quantitative data from heterogeneous literature. If not identified, such errors could propagate across interacting agents into downstream reasoning and ultimately misdirect experimental decisions. Future systems could mitigate these risks through confidence assessment, independent cross-checking of critical evidence, and traceable links between extracted information and its original sources.

The conceptual advance of this architecture lies not in the use of multiple AI agents but in its ability to organize heterogeneous scientific knowledge into a coordinated reasoning process. Conventional ML approaches generally rely on predefined descriptors and datasets to predict specific material properties, whereas scientific discovery often requires integrating heterogeneous knowledge, identifying critical problems, and balancing competing objectives. By linking literature analysis, numerical modeling, and molecular design within a unified reasoning process, the framework enables AI to move beyond task-specific prediction toward scientific reasoning, providing a foundation for AI-assisted hypothesis generation and complex materials discovery.

## Device co-design

The collaborative reasoning framework was further translated into an experimentally validated device co-design strategy (Figure 1C). In this process, the composition agent explored the FA-Cs compositional space through data-driven optimization to identify an optimal absorber composition. In parallel, the interface agent designed the ultraviolet-stable self-assembled monolayer MeO-DPPACz by extracting structure-property relationships from the literature. Compared with the widely used MeO-2PACz, the new molecule exhibits enhanced ultraviolet stability while maintaining favorable energy-level alignment for charge extraction. The central agent subsequently integrated these optimized design components with dual-side atomic-layer-deposited Al_2_O_3_ interlayers to formulate a complete inverted PSC architecture for experimental implementation.

The resulting device combines bulk defect control, interfacial stabilization, and ion-migration suppression within a unified design strategy, achieving a power conversion efficiency of 25.0% while retaining more than 97% of its initial efficiency after 1,000 h of continuous maximum-power-point operation at 100°C. More importantly, this strategy highlights a fundamental shift in photovoltaic materials discovery: the optimal device is not necessarily determined by the performance of individual components but by the compatibility and interactions among multiple functional layers. Future AI-driven photovoltaic design may therefore evolve from searching for superior materials to constructing optimized material ecosystems, where compositions, interfaces, and architectures are simultaneously tailored according to their coupled roles in device operation.

## Knowledge-evolving discovery

The most important contribution of the work by Guo et al. extends beyond the demonstration of a thermally stable PSC. An important feature of the proposed framework is its potential to support knowledge-evolving materials discovery, where the knowledge base available to each agent can be progressively expanded and refined as new literature and experimental results become available. Rather than relying solely on static datasets, this framework points toward more dynamic knowledge integration through retrieval-augmented generation (RAG). Unlike conventional AI models that are typically trained on fixed datasets and applied to predefined tasks, such a framework provides a pathway for AI systems to progressively refine their design strategies as validated scientific knowledge accumulates.

By integrating literature-derived insights, computational predictions, and experimental feedback within a unified workflow, the framework moves toward a more dynamic mode of AI-assisted discovery. In Guo et al., the curated literature dataset serves as an information retrieval pool, supporting a retrieval-augmented approach to agent-based reasoning. New observations can serve not only as validation results but also as additional sources of knowledge for improving future reasoning and design decisions. However, knowledge accumulation should not be equated with knowledge reliability: newly extracted information may contain errors and therefore requires validation before being incorporated into subsequent reasoning. Although fully autonomous knowledge updating remains an important future challenge, this concept provides a foundation for developing AI systems that continuously evolve alongside scientific progress.

The significance of this capability extends beyond improving predictive performance. Materials discovery is inherently an iterative process involving hypothesis generation, experimental validation, and knowledge refinement. Future systems could further combine retrieval-augmented reasoning with uncertainty-guided active learning, allowing informative or unexpected experimental results to trigger reevaluation of prior assumptions and guide subsequent experiments. Enabling AI systems to accumulate and reorganize scientific knowledge over time may ultimately transform AI from a static computational tool into a continuous learning partner for materials innovation.

## Concluding remarks

Looking forward, the integration of multi-agent reasoning with automated experimentation, high-throughput characterization, and intelligent laboratory platforms could enable a closed-loop paradigm for materials discovery, in which hypothesis generation, experimental validation, knowledge acquisition, and design refinement proceed continuously within a unified research workflow (Figure 1D).^4^ Such an adaptive framework may accelerate the discovery and optimization of complex materials by allowing AI-driven strategies to evolve with expanding experimental evidence rather than relying solely on predefined knowledge and datasets.

Within photovoltaics, this strategy is particularly promising for tandem solar cells, where absorber band gaps, recombination layers, current matching, and optical management must be optimized simultaneously.^5^ Beyond photovoltaics, similar challenges arise in perovskite light-emitting diodes, photodetectors, and other emerging optoelectronic systems governed by strong coupling among materials, interfaces, and device architectures. More broadly, the work by Guo et al. provides a blueprint for integrating AI with experimental research, pointing toward a future in which AI serves not only as a predictive tool but also as a continuously evolving partner in materials innovation.

## Funding and acknowledgments

This work is supported by the 10.13039/501100001809National Natural Science Foundation of China (62404185) and the 10.13039/501100021171Guangdong Basic and Applied Basic Research Foundation (2026A1515030023). J.Z. acknowledges support from the Nanqiang Outstanding Young Talents Program of Xiamen University (X2450215).

## Declaration of interests

The authors declare no competing interests.
